# The endotoxin-induced pulmonary inflammatory response is enhanced during the acute phase of influenza infection

**DOI:** 10.1186/s40635-018-0182-5

**Published:** 2018-07-05

**Authors:** R. M. Koch, D. A. Diavatopoulos, G. Ferwerda, P. Pickkers, M. I. de Jonge, M. Kox

**Affiliations:** 1 Department of Intensive Care Medicine, Radboud university medical centre, Nijmegen, The Netherlands; 2 Section Pediatric Infectious Diseases, Laboratory of Medical Immunology, Radboud Institute for Molecular Life Sciences, Radboud university medical centre, Nijmegen, The Netherlands; 3 Radboud Center for Infectious Diseases (RCI), Radboud university medical centre, Nijmegen, The Netherlands

**Keywords:** Influenza virus, LPS, Priming, Tolerance, Kinetics, Cytokines, MPO

## Abstract

**Background:**

Influenza infections are often complicated by secondary infections, which are associated with high morbidity and mortality, suggesting that influenza profoundly influences the immune response towards a subsequent pathogenic challenge. However, data on the immunological interplay between influenza and secondary infections are equivocal, with some studies reporting influenza-induced augmentation of the immune response, whereas others demonstrate that influenza suppresses the immune response towards a subsequent challenge. These contrasting results may be due to the use of various types of live bacteria as secondary challenges, which impedes clear interpretation of causal relations, and to differences in timing of the secondary challenge relative to influenza infection. Herein, we investigated whether influenza infection results in an enhanced or suppressed innate immune response upon a secondary challenge with bacterial lipopolysaccharide (LPS) in either the acute or the recovery phase of infection.

**Methods:**

Male C57BL/6J mice were intranasally inoculated with 5 × 10^3^ PFU influenza virus (pH1N1, strain A/Netherlands/602/2009) or mock treated. After 4 (acute phase) or 10 (recovery phase) days, 5 mg/kg LPS or saline was administered intravenously, and mice were sacrificed 90 min later. Cytokine levels in plasma and lung tissue, and lung myeloperoxidase (MPO) content were determined.

**Results:**

LPS administration 4 days after influenza infection resulted in a synergistic increase in TNF-α, IL-1β, and IL-6 concentrations in lung tissue, but not in plasma. This effect was also observed 10 days after influenza infection, albeit to a lesser extent. LPS-induced plasma levels of the anti-inflammatory cytokine IL-10 were enhanced 4 days after influenza infection, whereas a trend towards increased pulmonary IL-10 concentrations was found. LPS-induced increases in pulmonary MPO content tended to be enhanced as well, but only at 4 days post-infection.

**Conclusions:**

An LPS challenge in the acute phase of influenza infection results in an enhanced pulmonary pro-inflammatory innate immune response. These data increase our insight on influenza-bacterial interplay. Combing data of the present study with previous findings, it appears that this enhanced response is not beneficial in terms of protection against secondary infections, but rather damaging by increasing immunopathology.

## Background

Patients with influenza infection often suffer from severe secondary bacterial infections, which are associated with high morbidity and mortality rates [1, 2]. A striking example of this relationship was provided by bacteriological and histopathological analysis of infected lung tissue obtained from people who died of influenza during the 1918–1919 “Spanish flu” pandemic, in whom bacterial pneumonia was found to be the predominant cause of death [1]. These data suggest that an influenza infection profoundly influences the immune response upon a secondary bacterial infection.

Several studies have evaluated immunological interactions between influenza and bacterial infections, including infections with Gram-negative bacteria [3]. In vitro studies in which influenza-infected alveolar macrophages were subsequently stimulated with bacterial lipopolysaccharide (LPS), a bacterial compound that induces a profound innate immune response, revealed increased levels of pro-inflammatory cytokines tumor necrosis factor (TNF) α, interleukin (IL)-1β, and IL-6 [4–8], indicative of a priming effect on these cells by influenza. Data from in vivo animal studies are ambiguous. Similar to the in vitro data, some report enhanced responses. For instance, influenza infection in mice was shown to enhance the inflammatory response and neuropathogenicity resulting from LPS administration on days 3 and 4 after influenza inoculation [9]. Likewise, murine influenza infection resulted in increased levels of pro-inflammatory cytokines in both plasma and lungs, and enhanced pulmonary neutrophil influx upon pneumococcal infection 7 days later [10]. Similar results were observed in mice 14 days after influenza infection [11]. However, two otherwise largely comparable studies demonstrated reduced pulmonary pro-inflammatory cytokine concentrations upon *Streptococcus pneumoniae* and *Staphylococcus aureus* infections in mice infected with influenza 7 days before, indicative of influenza-induced immunosuppression [12, 13]. These equivocal results may be due to differences in the severity or kinetics of the influenza infection or the use of different bacteria as secondary challenges, thereby targeting various complex multi-receptor signaling pathways. Also, the use of live bacteria could have contributed to these ambiguous results. For instance, if influenza would induce immunosuppression and thereby facilitate outgrowth of bacteria upon a secondary live infectious challenge, the increased bacterial burden can eventually result in fulminant inflammation, which would wrongfully suggest influenza-induced augmentation of the immune response.

In the present study, we investigated whether influenza infection results in an enhanced or suppressed innate immune response upon a secondary challenge with LPS. Furthermore, we assessed the kinetics of these influenza-induced effects by performing the LPS challenges in either the acute or the recovery phase of influenza infection.

## Methods

### Ethics and animals

All procedures described were in accordance with the requirements of the Dutch Experiments on Animals Act, the EC Directive 86/609, and approved by the Animal Ethics Committee of the Radboud University Nijmegen Medical Center (RU-DEC 2013-029). Forty-eight male C57BL/6J mice (Charles River, Sutzfield, Germany) aged 10–12 weeks and weighing 23–29 g were used. Mice were housed in individually ventilated cages, with five mice per cage at the central animal facility of the Radboud University.

### Study design

At day 0, six groups of eight mice (total *n* = 48) were anesthetized by isoflurane and intranasally inoculated with a sublethal dose of influenza virus (pH1N1, strain A/Netherlands/602/2009, 5 × 10^3^ PFU) or mock treated (NaCl 0.9%) in a volume of 50 μL. Following infection, all mice were monitored and weighed daily. The temperature was recorded with an infrared thermometer on the skin, and physical condition was scored using a scoring and weight sheet (weight, body temperature, ruffled coat, hunched back, reduced mobility, and moribund). At either day 4 (acute phase) or day 10 (recovery phase), mice were placed in a temperature-controlled chamber to receive LPS (*E coli*, serotype 0111:B4, 5 mg/kg) or NaCl 0.9% by intravenous injection in the tail vein. Ninety minutes after LPS or NaCl administration, mice were deeply anesthetized with isoflurane and exsanguinated through orbital extraction, followed by cervical dislocation after which organs were collected.

### Blood and tissue collection

Ethylenediaminetetraacetic acid (EDTA)-anticoagulated blood was centrifuged at 13000×*g* for 2 min at room temperature after which plasma was stored at − 80 °C until analysis. Subsequently, perfusion of the lungs was performed by intracardiac injection with phosphate-buffered saline (PBS), after which lung lobes were harvested and snap frozen in liquid nitrogen and stored at − 80 °C until homogenization. Lung tissue was placed in 1 mL lysis buffer containing PBS, 0.5% triton X-100, and a protease inhibitor cocktail (complete EDTA-free tablets, Roche, Woerden, the Netherlands, 1 tablet per 50 mL lysis buffer). Subsequently, lung lobes were homogenized at 50 Hz, using a polytron homogenizer, and subjected to two rapid freeze-thaw cycles using liquid nitrogen. Finally, homogenates were centrifuged (10 min, 14,000×*g*, 4 °C), and the supernatant was stored at − 80 °C until cytokine analysis.

### Cytokine analysis

Concentrations of TNF-α, IL-1β, IL-6, and IL-10 in plasma and lung homogenates were measured using a Luminex assay (Milliplex, Millipore, Billerica, MA) according to the manufacturer’s instructions. The lower detection limit of the assay was 32 pg/mL for all cytokines. Plasma IL-1β levels were below the detection limit in the majority of animals. Lung homogenate cytokine concentrations were normalized to total protein content determined by bicinchoninic acid assay (BCA Protein Assay; Thermo Fisher Scientific).

### Myeloperoxidase content

Myeloperoxidase (MPO) content was measured in lung homogenates using an enzyme-linked immunosorbent assay (Hycult biotech, Uden, the Netherlands) according to the manufacturer’s instructions. Concentrations were normalized to total protein content as described above.

### Statistical analysis

All data were normally distributed according to the Shapiro-Wilk test. The Grubbs test (extreme studentized deviate method) was used to exclude significant outliers from analysis (maximum of one exclusion per dataset). To determine the number of animals required per group, we performed a power calculation based on a minimal detectable difference of 50% in LPS-induced plasma TNF-α levels between influenza-infected and non-influenza-infected mice. Mean ± SD (533 ± 163 pg/mL) TNF-α plasma levels were obtained from previous work from our group, in which male C57BL/6J mice were also injected intravenously with 5 mg/kg LPS and sacrificed 90 min later [14]. Using a two-sided α of 0.05 and a power of 80% (β of 0.2) in an unpaired *t* test design, six animals per group were required. To account for potential loss of animals due to influenza infection, eight animals per group were used. The effect size was based on previous work [9], in which influenza infection modulated the plasma cytokine response to LPS administration by at least 50%. Comparisons were analyzed using unpaired Student’s *t* tests and repeated measures one-way analysis of variance (ANOVA) as indicated in the figure legends. Statistical analyses were performed in GraphPad Prism 5.03 for Windows (GraphPad Software, San Diego, CA). Two-tailed *p* values < 0.05 were considered statistically significant.

## Results

### Clinical signs of influenza infection

**Fig. 1:**
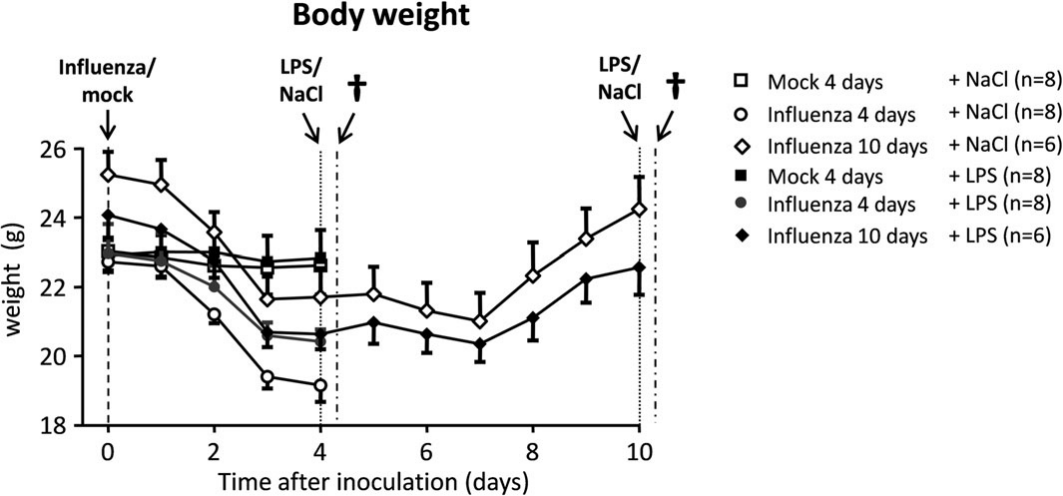
Body weight of influenza- or mock-inoculated mice. Data are presented as mean with SEM. Dagger indicates the two time points at which mice in the respective groups were sacrificed

All influenza-inoculated mice showed clinical signs of infection, including weight loss, lethargy, and pyrexia. Four influenza-infected mice were prematurely taken out of the experiment because of signs of severe infection. Body weight decreased in all influenza-infected mice in the acute phase of infection, whereas it remained stable in mock-inoculated mice (Fig. 1). From day 7 onwards, body weight started to increase, marking the recovery phase of influenza infection (Fig. 1).

### Cytokines in plasma and lung homogenates

**Fig. 2:**
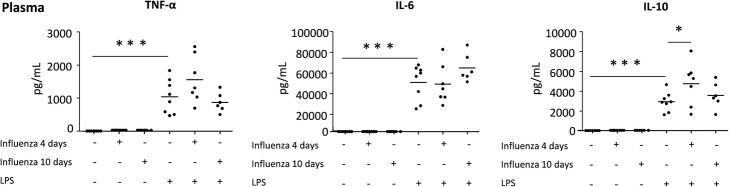
Plasma levels of TNF-α, IL-6, and IL-10 in mice that received influenza/mock followed by LPS/NaCl 4 or 10 days later. Data are presented as scatter-dot plots with horizontal lines indicating the mean value. *p < 0.05, **p < 0.01, ***p < 0.001 (calculated by unpaired Student’s *t* tests)

Influenza infection by itself did not result in increased plasma levels of any of the cytokines measured at both 4 and 10 days post-infection (Fig. 2). Expectedly, LPS administration led to profoundly increased plasma concentrations of TNF-α, IL-6, and IL-10. Although TNF-α and IL-6 plasma levels appeared to be somewhat higher in mice challenged with LPS 4 days after influenza infection compared with mock-inoculated mice, this did not reach statistical significance (*p* = 0.12 and *p* = 0.86, respectively). Plasma concentrations of the anti-inflammatory cytokine IL-10 were however significantly enhanced in mice challenged with LPS 4 days after influenza infection. No differences in any of the plasma cytokine levels were measured between influenza-infected and mock-inoculated mice at 10 days.

**Fig. 3:**
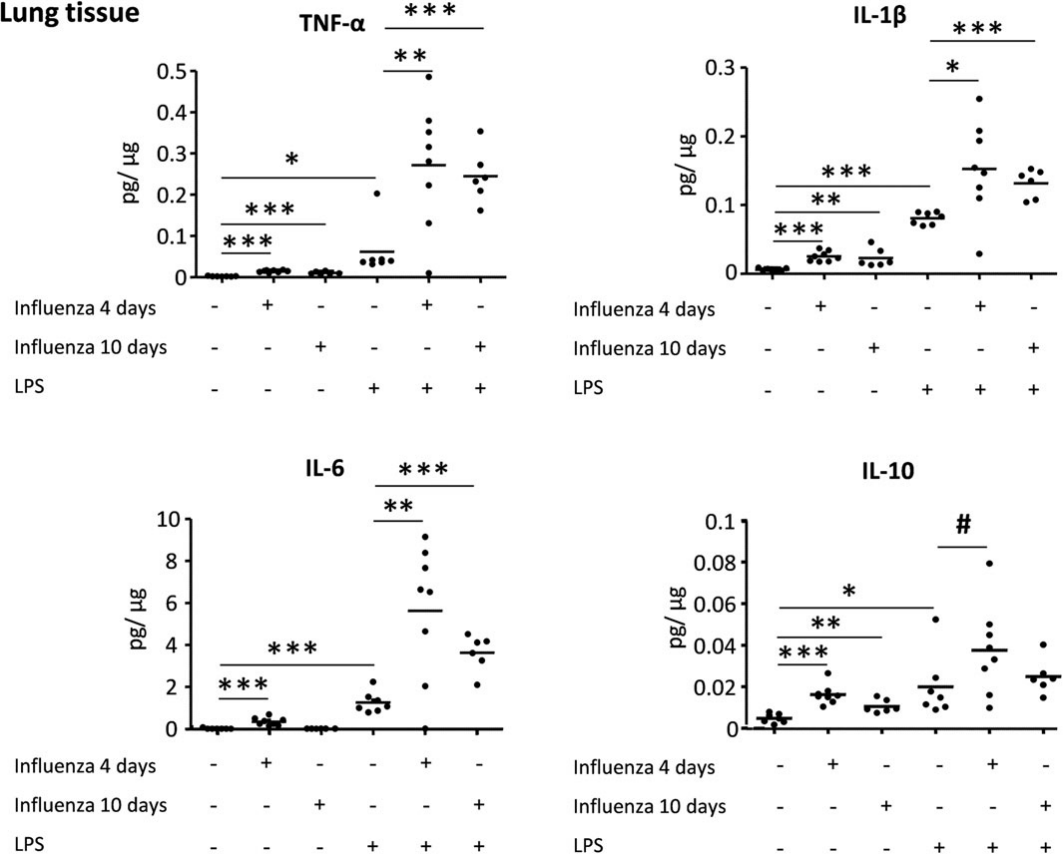
Levels of TNF-α, IL-1β, IL-6, and IL-10 in lung homogenates of mice that received influenza/mock followed by LPS/NaCl 4 or 10 days later. Data are presented as scatter-dot plots with horizontal lines indicating the mean value. *p < 0.05, **p < 0.01, ***p < 0.001, ^#^*p* = 0.05–0.10 (calculated by unpaired Student’s *t* tests)

In lung homogenates, influenza by itself caused mildly elevated levels of TNF-α, IL-1β, IL-6, and IL-10 at 4 days post-infection and to a lesser extent at 10 days after infection (Fig. 3). Similar to what was found in plasma, LPS challenge also led to increased concentrations of all measured cytokines in lung tissue. A synergistic increase of all pro-inflammatory cytokines in the lungs was found in influenza-infected mice challenged with LPS 4 days later and, to a lesser extent, in mice challenged with LPS 10 days post-influenza infection. For IL-10, the potentiating effect was additive rather than synergistic, only observed at 4 days post-influenza infection, and reached a trend towards statistical significance.

### Pulmonary MPO content

**Fig. 4:**
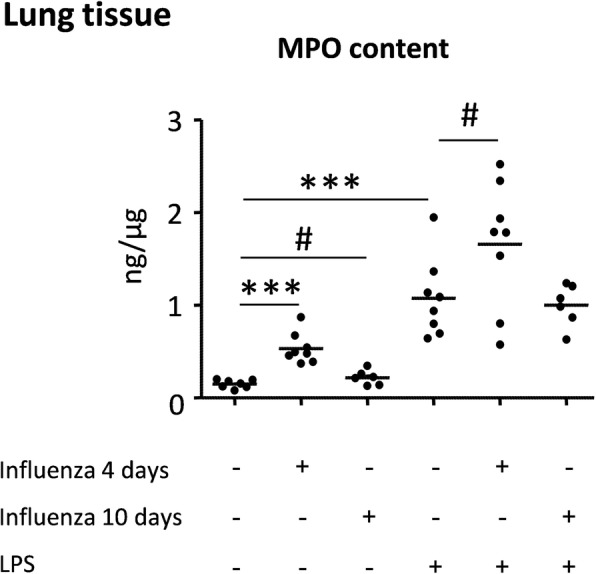
MPO content in lung homogenates of mice that received mock/influenza followed by NaCl/LPS 4 or 10 days later. Data are presented as scatter-dot plots with horizontal lines indicating the mean value. *p < 0.05, **p < 0.01, ***p < 0.001, ^#^*p* = 0.05–0.10 (calculated by unpaired Student’s *t* tests)

In accordance with pulmonary cytokine levels, influenza infection by itself led to increased MPO content in the lungs 4 days after infection and tended to result in increased MPO content 10 days post-infection (Fig. 4). Again, LPS administration also resulted in increased MPO levels in lung tissue, and there was a trend towards enhanced MPO content in influenza-infected mice challenged with LPS 4 days after infection.

## Discussion

In the present study, we demonstrate that a systemic LPS challenge in the acute phase of influenza infection (4 days post-infection) results in an enhanced pulmonary, but not systemic pro-inflammatory cytokine response. This effect was synergistic rather than additive, indicating that influenza infection actually modulates the immune response to a subsequent challenge with LPS. Furthermore, this effect remained present, although less pronounced, in the recovery phase of influenza infection (10 days post-infection). The LPS-induced increase in MPO content in lung homogenates, reflecting pulmonary neutrophil influx or sequestration, tended to be enhanced in the acute phase of influenza infection as well. These results suggest that influenza infection, especially in the acute phase, may cause a more pronounced pulmonary pro-inflammatory immune response upon a secondary bacterial infection.

Our results are in accordance with in vitro data reporting a cellular priming effect of influenza observed upon secondary stimulation with LPS [4–8], as well as with other murine in vivo studies that report increased inflammation and pulmonary neutrophil influx or sequestration upon a secondary bacterial infection or LPS challenge in the acute phase of influenza infection [9, 10]. For example, a preceding influenza infection in mice gravely enhanced lung injury induced by a secondary infection with *Streptococcus pneumoniae* 7 days later, resulting in a severe necrotic pneumonia accompanied by increased mortality [10]. Also, the increased MPO content observed in our study is an important hallmark of acute respiratory distress syndrome (ARDS) [15, 16], a severe complication of influenza infection caused by excessive pulmonary inflammation. These and our study reveal that the enhancing effect on the pro-inflammatory innate immune response is most evident in the lungs, probably because the influenza-induced damage and consequent inflammatory effects are most pronounced at this site. In this context, our data are in line with the recommendation to use corticosteroids in patients with severe influenza infections in the Intensive Care Unit to counteract the pulmonary hyperinflammatory response causing ARDS. Several underlying mechanisms may contribute to the observed effects. At the cellular level, studies have shown that influenza and certain bacterial pathogens, such as *Haemophilus influenzae* and *Streptococcus pneumoniae*, utilize similar immunological pathways and that the overlap in the inflammatory mediators produced thereby creates augmentation of the immune response during sequential infection, in turn causing immunopathology [17, 18]. Furthermore, it has been hypothesized that influenza stimulates TNF-α gene transcription activators or may interfere with labile transcription repressor proteins and stabilizes TNF-α mRNA by delaying its degradation [8]. Alternatively, the increased lung MPO levels observed do not necessarily reflect PMN infiltration into the lungs, but may (also) result from PMNs trapped in the vasculature, as circulating activated neutrophils become rigid and can be trapped within the small capillaries of the lung [19]. As such, increased entrapment of leukocytes in the pulmonary vasculature during influenza infection could also contribute to the enhanced inflammatory cytokine levels upon LPS challenge. We can only speculate on this, because no histological data are available, which represents a limitation of this work.

It may be argued that the enhanced pro-inflammatory immune response induced by influenza serves as a means to efficiently eliminate the primary pathogen and to enhance host defense towards a secondary infection. For instance, pro-inflammatory cytokines are induced in influenza-infected cells to limit viral replication and to initiate downstream immune responses [20]. However, this is not supported by previous work, where an increased bacterial burden was observed irrespective of an enhanced or suppressed response [11–13]. Several explanations for this observation may be put forward. First, next to potentiating pro-inflammatory cytokine responses, the present study and work by others [11] have shown that influenza infection also potentiates production of the key anti-inflammatory cytokine IL-10, which was demonstrated to be crucial in facilitating bacterial outgrowth upon secondary challenge with *Streptococcus pneumoniae* [11]. Second, influenza may on the one hand prime for production of innate cytokines produced by myeloid cells, but impair T cell-derived cytokines that are instrumental for the adaptive immune response. This was elegantly demonstrated by Kudva et al., who showed that, in line with our results, infection with *Staphylococcus aureus* 6 days after influenza resulted in increased pulmonary levels of innate cytokines such as IL-6 and MCP-1, and increased neutrophil influx to the lungs, but decreased concentrations of T-cell-derived IL-17 and IL-22, which were demonstrated to play a pivotal role in fending off the staphylococcal infection [21].

Whereas the enhancing effects of influenza on pro-inflammatory innate immune parameters were less pronounced at 10 days post-infection, a suppressed response was neither evident. This could be partly biased by the exclusion of two mice in both recovery groups due to severe influenza infection. However, it might also be argued that 10 days post-infection is too soon for these effects to manifest. For example, profound desensitization towards LPS and flagellin, another Toll-like receptor (TLR) ligand, was observed in alveolar macrophages obtained from mice up to 6 weeks after influenza infection [22]. Furthermore, the direction of the response upon a secondary challenge is probably highly dependent on the pathogen or stimulus used, each using distinct intracellular signaling pathways. With regard to this, it is well-known that influenza virus particularly predisposes to *Aspergillus fumigatus*, which is present in 25% of all influenza patients [23, 24], causing infections such as invasive pulmonary aspergillosis that are associated with very high mortality rates. As different mechanisms may be important in host defense towards various pathogens, the specific response towards *Aspergillus* *fumigatus* could be suppressed by a preceding influenza infection. The use of corticosteroids may be another important factor in the observed vulnerability towards particular secondary infections, as steroid use was shown to be independently associated with the presence of *Aspergillus fumigatus* in sputum of cystic fibrosis patients [25] and with a substantially increased risk of community-acquired *Staphylococcus aureus* bacteremia [26]. Furthermore, a meta-analysis revealed that the use of corticosteroids was significantly associated with nosocomial infections [27]. To the best of our knowledge, these putative detrimental effects of corticosteroid treatment during influenza on secondary infections have yet to be studied systematically in animal models. In any case, it remains to be determined whether the overall effects of corticosteroid treatment are beneficial or not, as they may lead to increased susceptibility in a subset of influenza virus-infected patients but may also provide health benefits in another subset of influenza virus-infected patients.

## Conclusions

An LPS challenge in the acute phase of influenza infection results in an enhanced pulmonary pro-inflammatory innate immune response. These data increases our insight concerning viral-bacterial interplay. Combined with previous findings, it appears that this enhanced pro-inflammatory response does not lead to protection against secondary infections but rather causes immunopathology leading to damage, and thereby to organ failure.

## Abbreviations

ANOVA: One-way analysis of variance
ARDS: Acute respiratory distress syndrome
BCA: Bicinchoninic acid assay
IL: Interleukin
LPS: Lipopolysaccharide
MPO: Myeloperoxidase
TNF: Tumor necrosis factor
